# Cortical morphological changes in stroke-associated dysarthria patients

**DOI:** 10.3389/fneur.2026.1766268

**Published:** 2026-03-19

**Authors:** Jin Zhou, Shilong Zhao, Wenjing Zhang, Tianyuan Wei, Hongxia Zhang, Weijin Liu, Chunlin Li, Xiaoxia Du

**Affiliations:** 1 Department of Neurorehabilitation, China Rehabilitation Research Center, Beijing, China; 2 China Rehabilitation Science Institute, Beijing, China; 3 School of Rehabilitation Medicine, Capital Medical University, Beijing, China; 4 School of Biomedical Engineering, Capital Medical University, Beijing, China; 5 Beijing Stomatological Hospital, Capital Medical University, Beijing, China; 6 Department of Rehabilitation, First Affiliated Hospital of Xi’an Jiaotong University, Xi’an, China; 7 Department of Radiology, Beijing Bo'ai Hospital, China Rehabilitation Research Center, Beijing, China

**Keywords:** cortical structure, dysarthria, fractal dimension, gyrification index, stroke

## Abstract

**Objective:**

Post-stroke dysarthria (DYS) severely affects communication, yet cortical morphological alterations remain insufficiently characterized. This study applied surface-based morphometry (SBM) to examine cortical changes in DYS and their association with dysarthria severity assessed by the Frenchay Dysarthria Assessment (FDA).

**Methods:**

Forty-eight DYS patients and 72 matched controls underwent MRI. Cortical fractal dimension and gyrification were extracted via SBM. Group differences were tested using two-sample *t*-tests, and correlations with FDA scores were assessed using Spearman analyses.

**Results:**

DYS patients exhibited significantly increased fractal dimension in the left insula and elevated gyrification in bilateral insula, superior temporal and precentral gyri (*p* < 0.05). Right supramarginal gyrification positively correlated with FDA reflex and respiration subscores (*ρ* > 0.3, *p* < 0.05). No significant differences were observed between supratentorial and infratentorial lesions.

**Conclusion:**

Cortical morphological alterations in insula and supramarginal gyrus may contribute to DYS pathophysiology. SBM provides quantitative markers linking cortical architecture to speech motor control, potentially guiding individualized rehabilitation strategies.

## 1 Introduction

Stroke is a leading cause of death and long-term disability among adults worldwide. In China, there are 28.76 million stroke patients, with the incidence continuing to rise annually, hence becoming a significant public health challenge (1). Dysarthria, a common neurogenic motor disorder following stroke, primarily results from impairments in the speed, strength, accuracy, range, pitch, or duration necessary for speech control (2, 3). Previous studies have indicated that approximately 52% of stroke survivors have some level of dysarthria (4). Compared with stroke patients without dysarthria, affected individuals generally echibit poorer overall health status, reduced mental wellbeing, and a higher risk of social isolation, which substantially compromises communication abilities and quality of life (5). In addition, dysarthria does not only rise social and functional impairments, but it is also a major economic burden for families and society (6).

Speech production is a distinctive higher-order neural function in humans, constituting a complex and coordinated interaction of sensory, motor, and cognitive processes. Beyond the classical language areas, speech production also involves multiple brain regions responsible for motor control, auditory feedback, sensory processing, and cognitive integration (7). The post-stroke dysarthria (DYS) researches focuses on factors such as gray matter volume and infarct topography (8). Previous studies have identified key brain regions associated with stroke-caused DYS, including the supratentorial region (such as the motor cortex, middle cerebral artery territory, thalamus, basal ganglia) and infratentorial region (cerebellum, midbrain) (9, 10). However, despite growing interest in the neural mechanisms underlying speech prodiction, structural brain changes specifically associated with post-stoke DYS remain insufficiently characterized, particularly at the level of cortical morphology.

Surface-based morphometry (SBM) provides a more detailed characterization of cortical structure than traditional gray matter volume measures by quantifying features such as cortical thickness, surface area, gyrification, and cortical curvature (11–13). These measures are more sensitive in detecting subtle microstructural changes in local brain tissue post-stroke, thereby offering a more accurate reflection of the neurobiological processes underlying DYS. Studies have demonstrated that the severity of dysarthria in Parkinson’s disease (PD) correlates significantly with cortical thickness in the precentral gyrus and fusiform gyrus (14). However, systematic investigation of cortical morphological changes in post-stroke DYS are still lacking, particularly for speech production crucial regions, such as the insula and supramarginal gyrus, where the specific patterns of morphological alterations in these areas remain unknown.

So far, the cortical morphological features of stroke-induced DYS remain poorly understood, particularly regarding the structural changes in the cortex associated with speech impairment, which have not been systematically investigated. Moreover, the current measures of assessing DYS are mostly subjective rating scales, while objective neuroimaging-based markers remain exploratory.

Therefore, the present study was an exploratory, hypothesis-generating investigation using SBM to characterize cortical morphological differences between stoke patients with dysarthria and healthy subjects. Specifically, we examined fractal dimension and gyrification index alterations and explored their associations with clinical severity as assessed by the FDA. Rather than inferring causality or clinical applicability, this study aims to provide descriptive structural evidence that may inform future longitudinal and mechanistic research on post-stroke dysarthria. To address these gaps in research, the current research is aimed to compare systematically the cortical morphological differences between DYS patients and healthy controls through SBM. The focus will be on investigating changes in subcortical gray matter structures and analysing the correlations between morphological parameters and scores from the Frenchay Dysarthria Assessment (FDA). The purpose is to provide new insights into neuropathological processes of DYS. The findings of this study will assist in bridging the gaps in cortical morphology of post-stroke DYS and offer new neuroimaging-based evidence for guiding more accurate assessments and specific rehabilitation protocols in the future.

## 2 Materials and methods

### 2.1 Participants

The cross-sectional research was conducted from January 2020 to December 2024. The subjects were 48 stroke-related dysarthria patients from the Neurological Rehabilitation Center of China Rehabilitation Research Center. Among the 48 participants, there were 39 males and 9 females, all of Han ethnicity, with an average age of 55.38 years. Among them, 14 patients were diagnosed with supratentorial stroke, and 23 patients were diagnosed with infratentorial stroke. Inclusion criteria included: (1) post-stroke DYS patients, within 1 week to 1 year after onset; (2) 18–80 years of age; (3) native Chinese speakers, with fluency in Mandarin; (4) clinically diagnosed as DYS; (5) right-handed; (6) no contraindications for MRI examination; (7) normal or corrected-to-normal vision, and no special growth or educational background; (8) no history of speech rehabilitation therapy; (9) no psychiatric history or history of substance abuse. Exclusion criteria included: (1) severe consciousness or cognitive impairments; (2) severe comorbidities (e.g., heart failure, renal failure, cancer, etc.); (3) combined sensory or auditory impairments; (4) stuttering or aphasia history; (5) being unable to cooperate with the assessment process; (6) refusal to participate in the study. A healthy control group of 72 participants was recruited from the general population, matched for age and gender with the patient group, including 46 males and 26 females, with a mean age of 50.49 years. In the patient group, the average time interval between ischemic stroke onset and MRI examination was 143.46 days. All enrolled participants were clinically assessment through standardized scales by clinicians and speech therapists with over 3 years of relevant experience. The MRI scanning procedure was performed by trained radiologists with more than 3 years of experience in MRI scanning. This study was approved by the Medical Ethics Committee of China Rehabilitation Research Center (Approval No. CRRC-IEC-RF-SC-005-01).

### 2.2 Clinical data

This research collected extensive clinical information from all participants, including demographic data, the duration between stroke onset or the recent clinical visit and the MRI scan, as well as scores from the Frenchay Dysarthria Assessment (FDA). The FDA is a widely used and practical tool for the assessment of stroke patients with dysarthria, particularly in China, where it has become a standard clinical instrument (15). By the evaluation of a broad range of domains, the FDA allows clinicians to comprehensively evaluate speech and swallowing impairments, identify specific deficits, and track recovery progress. A total of 43 patients were evaluated using the FDA, which is a validated tool for measuring dysarthria. The FDA consists of eight sections, each designed to assess various aspects of oral structures and speech functions, such as reflexes, respiration, lips, jaw, palate, voice, tongue, and speech intelligibility (16).

### 2.3 MRI data acquisition

MRI data was obtained from the BoAi Hospital, Beijing, China, and an MRI system at 3.0 T (GE, SIGNA EXCITE) was applied. The following MRI parameters of the 2D T1-weighted inversion recovery (IR) sequence were recalled in axial orientation: repetition time (TR) = 2259.12 ms, echo time (TE) = 11.272 ms, flip angle (FA) = 90°, field of view (FOV) = 230 × 208 mm^2^, acquisition matrix = 256 × 201, voxel size = 0.9 × 1 × 5 mm^3^, slice number = 23 slices, slice thickness = 5.0 mm.

### 2.4 Data processing

The surface-based analysis was performed using the Computational anatomy toolbox (CAT12),^1^ which is an automated approach based on a projection-based thickness (PBT) method to process fractal dimension and gyrification. We used recommended default options. We used recommended default options (17). The specific process is as follows: 1. pre-processing: cortical surface estimation, topological correction, and spherical mapping or registration; 2. index extraction: fractal dimension and gyrification were resampled and smooth surfaces by using 20 mm full width at half maximum (FWHM) Gaussian kernel, respectively.

### 2.5 Statistical analyses

Demographic and clinical data were analyzed using Python. Statistical significance was set at *p* < 0.05. The normality of quantitative variables was checked using the Shapiro–Wilk test. For data satisfying normality, two-sample *t*-test or analysis of covariance (ANCOVA) were used for testing. For data not satisfying normality, rank based analysis of covariance (rank based ANCOVA) or Mann–Whitney *U* test were used for testing. Age, gender and duration of illness were used as covariates in the analysis of covariance to compare the FDA scale between two groups of stroke patients. Gender was compared using chi-square test.

The cortical morphological analyses were performed using two-sample *t* test to compare differences between patients with stroke and HCs by utilizing age and sex as covariates, we also compare differences between patients with supratentorial stroke and patients with infratentorial stroke, and cluster-level family-wised error (FWE) correction was used for multiple comparisons (uncorrected vertex *p* < 0.001, cluster *p* < 0.05).

In addition, based on the results of the surface-based morphometry, we estimated the spearman correlation between the mean cortical characteristics and scores on each component of the FDA, and Benjamini-Hochberg False Discovery Rate (BH-FDR) correction is used to perform multiple comparison correction on statistical results of correlation (*q* < 0.05, two tailed), and Benjamini-Hochberg False Discovery Rate (BH-FDR) correction is used to perform multiple comparison correction on statistical results of correlation (*q* < 0.05, two tailed).

## 3 Results

### 3.1 Demographics and clinical measures

A total of Table 1 presents the demographic and clinical characteristics of the stroke patients and healthy controls (HCs). The mean age of the stroke patients was 55.38 ± 13.23 years, while the mean age of the HCs was 50.49 ± 13.92 years, with no significant difference between the two groups (*p* = 0.07, using a two-sample *t*-test). Regarding gender distribution, there were 9 females and 39 males in the patient group, compared to 26 females and 46 males in the HC group. The difference in gender distribution between the two groups was also not statistically significant (*p* = 0.06, using a Chi-square test). These results suggest that the two groups were well-matched in terms of age and gender, with no significant demographic differences that could influence the outcomes of the study.

**Table 1:** Demographic and clinical data between Stroke Patients and HCs.

Variable	Stroke patients (*n* = 48)	HCs (*n* = 72)	*T*/*X*^2^	*p*
Age	55.38 ± 13.23	50.49 ± 13.92	1.91	0.06^a^
Gender (female/male)	9/39	26/46	3.40	0.07^b^

a Two-sample *t* test.

b Chi-square test.

**Figure 1:**
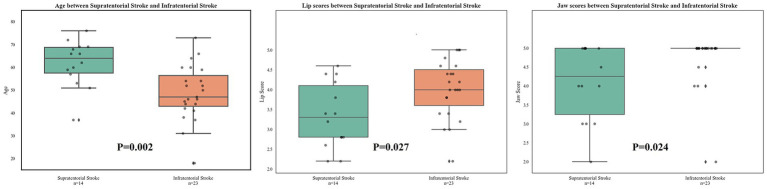
Significant differences in FDA score between supratentorial stroke group and infratentorial stroke group. FDA, The Frenchay Dysarthria Assessment.

In Figure 1 and Table 2, we found that patients with infratentorial stroke were significantly younger than those with supratentorial stroke, and patients with infratentorial stroke scored significantly higher on the lip and jaw portions of the FDA scale than patients with supratentorial stroke.

**Table 2:** Demographic and clinical data between supratentorial stroke and infratentorial stroke.

Variable	Supratentorial stroke (*n* = 14)	Infratentorial stroke (*n* = 23)	*T*/*U*/*F*/*X*^2^	*p*-value
Age, years	61.79 ± 9.73	48.83 ± 11.89	3.34	**0.002** ^**a**^^,*^
Gender (female/male)	2/12	7/16	0.51	0.47^e^
Disease duration (month)	4.50 ± 2.32	5.70 ± 3.25	124.0	0.250^b^
FDA score	28.57 ± 6.28	32.25 ± 5.40	1.89	0.179^c^
Reflexes	3.64 ± 0.89	4.10 ± 0.78	0.93	0.341^d^
Respiration	3.54 ± 1.06	4.15 ± 0.90	1.34	0.256^d^
Lips	3.34 ± 0.79	4.02 ± 0.71	5.4	**0.025** ^c*^
Jaw	4.11 ± 0.97	4.76 ± 0.66	5.66	**0.024** ^d*^
Palate	4.17 ± 0.64	4.23 ± 0.85	0.02	0.896^d^
Voice	3.07 ± 0.86	3.46 ± 0.91	0.69	0.411^c^
Tongue	3.38 ± 0.79	3.82 ± 0.74	2.33	0.137^c^
Speech intelligibility	3.32 ± 1.29	3.71 ± 0.95	0.85	0.364^c^

FDA, the Frenchay Dysarthria Assessment.

a Two-sample *t* test.

b ANCOVA.

c ANCOVA.

d Rank based ANCOVA.

e Chi-square test. *indicate statistical significance at *p* < 0.05. Bold font is used to highlight the significant values.

### 3.2 Fractal dimension comparisons between the groups

**Figure 2:**
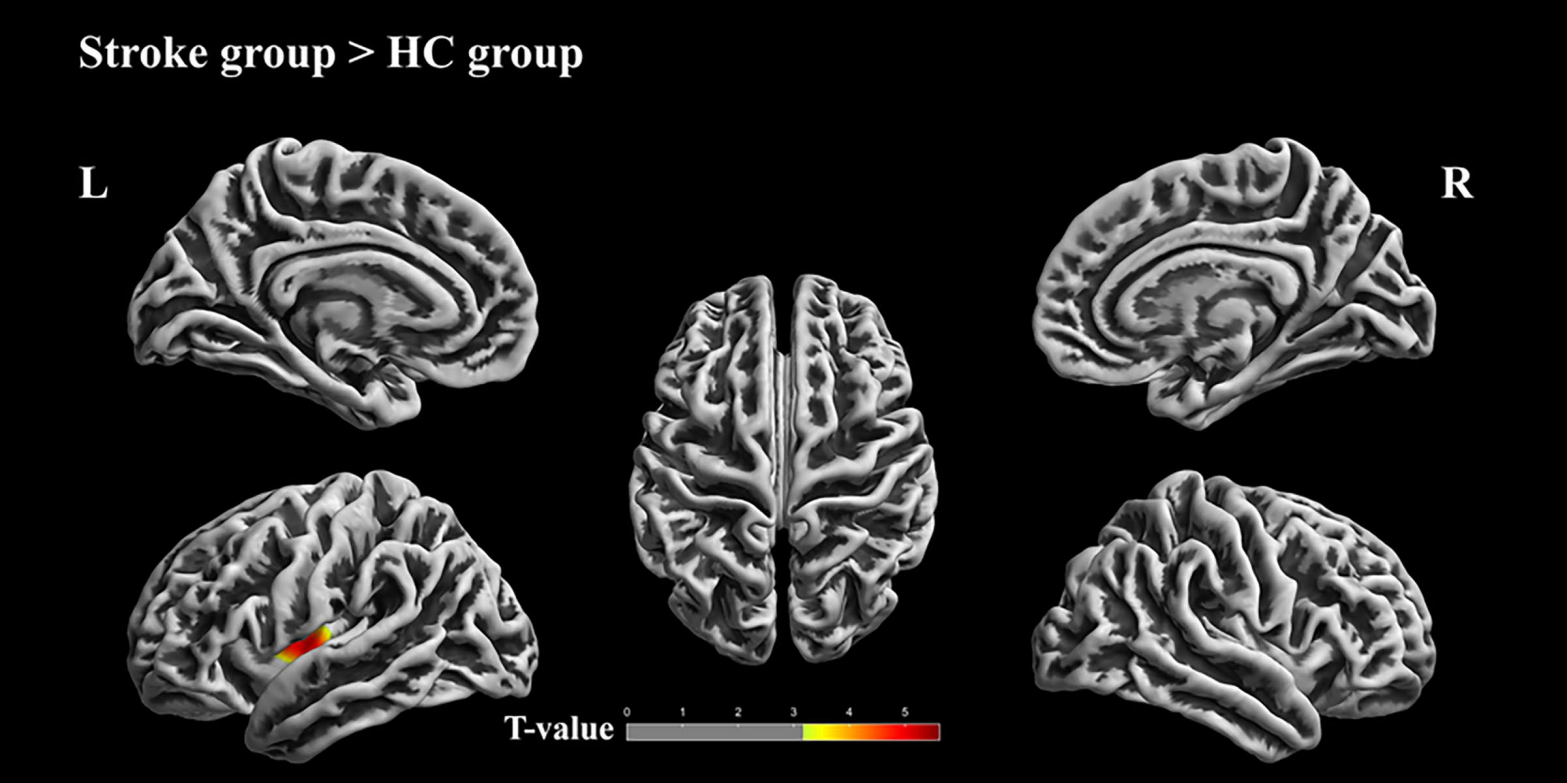
Differences in fractal dimension between the patients with stroke and HCs. Results of two-sample *t* test; cluster-level family-wised error (FWE) correct, uncorrected vertex *p* < 0.001, cluster *p* < 0.05. The color bar represents *t* values. Warm colors indicate that values in stroke patients are greater than those in HCs. L, Left hemisphere; R, Right hemisphere.

Figure 2 and Table 3 present the fractal dimension difference between the patients with stroke and HCs evaluated using two-sample *t* test, with age and sex as covariates. Patients with stroke exhibited increased fractal dimension in the left insula cortex (uncorrected vertex *p* < 0.001, cluster *p* < 0.05, cluster-level FWE correction).

**Table 3:** Significant clusters showing group differences in fractal dimension (Cluster-level family-wised error (FWE) correction, uncorrected vertex *p* < 0.001, cluster *p* < 0.05).

Group	Brain region	Cluster size	*T*	Uncorrected *p*
Stroke > HCs	100%left insula	253	5.10	0.000

We found no differences between patients with supratentorial stroke and patients with infratentorial stroke.

### 3.3 Gyrification comparisons between the groups

**Figure 3:**
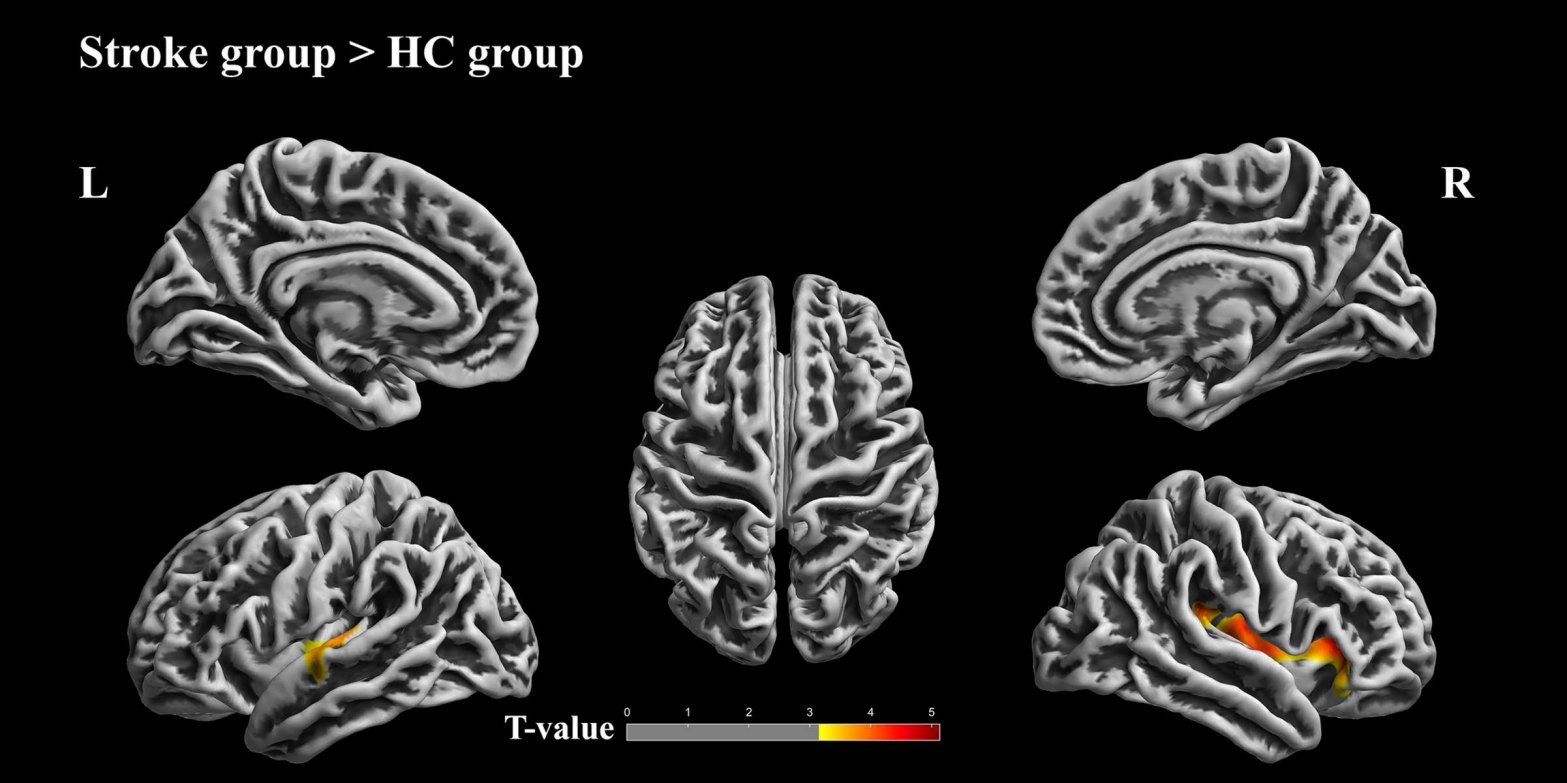
Differences in gyrification between the patients with stroke and HCs. Results of two-sample *t* test; cluster-level family-wised error (FWE) correct, uncorrected vertex *p* < 0.001, cluster *p* < 0.05. The color bar represents *t* values. Warm colors indicate that values in stroke patients are greater than those in HCs. L, Left hemisphere; R, Right hemisphere.

In Figure 3 and Table 4, the patients with stroke exhibited higher gyrification in some regions, namely left superior temporal, left transverse temporal, left insula, right insula, right supramarginal, right superior temporal, right postcentral, right precentral, right lateral orbitofrontal, right pars triangularis, right transverse temporal, right pars orbitalis than HCs(uncorrected vertex *p* < 0.001, cluster *p* < 0.05, cluster-level FWE correction).

**Table 4:** Significant clusters showing group differences in gyrification (Cluster-level family-wised error (FWE) correction, uncorrected vertex *p* < 0.001, cluster *p* < 0.05).

Group	Brain region	Cluster size	*T*	Uncorrected *p*
Stroke > HCs	48% left superior temporal 28% left transverse temporal 25% left insula	420	4.20	0.000
Stroke > HCs	45% right insula 14% right supramarginal 9% right superior temporal 8% right postcentral 6% right precentral 6% right lateral orbitofrontal 5% right pars triangularis 4% right transverse temporal 2% right pars orbitalis	1,515	4.70	0.000

We found no differences between patients with supratentorial stroke and patients with infratentorial stroke.

### 3.4 Results of the correlation analysis

**Figure 4:**
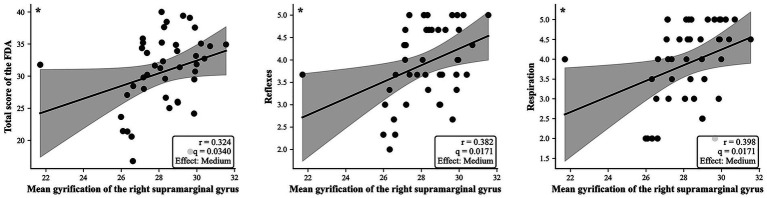
Spearman correlation analyses exhibited that the gyrification increased in the right supramarginal gyrus of patients with stroke and demonstrated correlation with total score of the FDA, reflexes, and respiration.

In Figure 4, we observed a significant positive correlation between the mean gyrification of the right supramarginal gyrus and the total score on the FDA (*r* = 0.324, *q* = 0.0340, Effect: Medium, two tailed). Additionally, a significant positive correlation was found between the mean gyrification of the right supramarginal gyrus and reflexes (*r* = 0.382, *q* = 0.0171, Effect: Medium, two-tailed). Furthermore, the mean gyrification of the right supramarginal gyrus was also significantly and positively correlated with respiration (*r* = 0.398, *q* = 0.0171, Effect: Medium, two-tailed).

## 4 Discussion

In this study, SBM was applied to explore cortical morphological characteristics in stroke patients with dysarthria and their associations with clinical speech performance. Compared with healthy controls, patients exhibited altered fractal dimension and gyrification in several cortical regions, particularly the insula and supramarginal gyrus. These findings should be interpreted as descriptive and exploratory, reflecting potential structural correlates of post-stoke dysarthria rather than definitive neural mechanisms.

Through the analysis of fractal dimension and the gyrification index, this current work provides an initial characterization of cortical surface features in post-stoke dysarthria and contributes a preliminary contribution to a relatively underexplored area of neuroimaging research. The results primarily generate hypotheses regarding post-stoke cortical reorganization that warrant further confirmation in longitudinal and multimodal studies.

### 4.1 Functional differences between supratentorial and infratentorial stroke patients

In this study, we compared the epidemiologic, FDA scale, and brain structural data between patients with supratentorial stroke and infratentorial stroke to explore functional differentitation between speech and swallowing functions. The supratentorial stroke group was significantly older than the infratentorial group. This finding aligns with previous studies, which suggest that infratentorial strokes are observed to affect younger individuals, primarily affecting the pons and brainstem (18), whereas supratentorial strokes are more prevalent in older populations and affect cortical and subcortical areas (19).

The FDA scores showed higher lip and jaw function scores in the infratentorial group, which may reflect relatively greater involvement of cortical motor regions in supratentorial stoke. No significant group differences were observed in other FDA domains, including reflexes, respiration, palate, voice, tongue function, or speech intelligibility. Although total FDA scores tended to be higher in the infratentorial group, this difference did not reach statistical significance, suggesting broadly comparable overall speech performance.

No significant differences in cortical surface morphology were detected between supratentorial and infratentorial stroke groups. This finding should be interpreted cautiously, as subgroup sample sizes were limited and SBM primarily captures cortical features, potentially reducing sensitivity to brainstem or cerebellar pathology common in infratentorial stroke. In addition, shared network-level adaptive processes across stroke locations may obscure location-specific cortical differences.

### 4.2 Changes in fractal dimension in stroke patients with DYS

Stroke patients with dysarthria exhibited increased fractal dimension in the left insular cortex. Fractal dimension reflects cortical surface complexity and has been linked to structural organization. The insula plays a critical role in sensorimotor integration for speech production and swallowing (20, 21).

Given prior evidence linking insular damage to dysarthria and dysphagia, the observed incease in fractal dimension may reflect post-stroke structural reorganization within a region central to speech motor control. Such changes may be related to adaptive or compensatory processes following stoke, although alternative interpretations, including nonspecific remodeling, cannot be excluded. These findings are consistent with previous studies suggesting a potential role of the insula in post-stroke speech recovery.

### 4.3 Changes in gyrification index in stroke patients

The GI reflects cortical folding complexity and has been associated with brain structural organization and network integration (22, 23). While reduced GI has been reported in neurodegenerative conditions such as Alzheimer’s disease, the present study identified increased gyrification in several cortical regions in stroke patients, including the bilateral insula, superior temporal gyrus, transverse temporal gyrus, and right supramarginal gyrus,

These regions are involved in auditory processing, language comprehension, phonological encoding, and sensorimotor integration (24–26). Increased GI in these areas may reflect cortical reorganization within speech-related networks following stoke. Rather than indicating functional enhancement, such changes are more appropriately interpreted as structural adaptations associated with altered neural demands.

### 4.4 Associations between cortical morphology and clinical scores

Exploratory correlation analyses revealed positive associations between GI of the right supramarginal gyrus and FDA reflexes, respiration, and total scores. The supramarginal gyrus plays an important role in phonological processing and sensory-motor integration during speech and swallowing (27, 28).

These associations suggest that cortical morphological characteristics of the right supramarginal gyrus may be related to specific aspects of speech and swallowing performance. Increased gyrification in this region may reflect reorganization within neural networks supporting reflexive swallowing, respiratory control, and coordination between breathing and speech (28–30). However, due to the cross-sectional design, causality and directionality cannot be determined, and these findings should be regarded as preliminary.

## 5 Conclusion

This research conducted a SBM analysis of stroke patients with dysarthria, which revealed for the first time cortical morphological alterations that correlate with dysarthria in stroke patients. Specifically, alterations in fractal dimension and gyrification were observed in several speech-related cortical regions, particularly the insula and right supramarginal gyrus, and exploratory associations were identified between gyrification measures and clinical speech performance assessed by the Frenchay Dysarthria Assessment,

These findings provide preliminary structural evidence that post-stroke dysarthria may be accompanied by cortical surface reorganization within networks involved in speech production, sensory integration, and motor control. Increased fractal dimension and gyrification may reflect adaptive or compensatory morphological changes following stroke (31). Moreover, SBM such as gyrification and fractal dimension have been shown to be sensitive to experience-dependent cortical plasticity, supporting their potential relevance as markers of adaptive reorganization their functional significance and clinical relevance require further investigation (32).

From a clinical perspective, the identification of speech-related cortical surface alterations may contribute to a better neuroanatomical understanding of post-stroke dysarthria and may, in the future, inform the development of imaging-based markers for monitoring speech impairment and recovery. Overall, this study contributes descriptive neuroimaging data to an underexplored area of post-stroke dysarthria research. Consistent with recent reviews of MRI-based neuroplasticity research in stroke, Future longitudinal and multimodal studies are needed to clarify the temporal dynamics, underlying mechanisms, and potential clinical value of cortical surface morphological changes in speech recovery after stroke (33).

## 6 Limitation

This study has the following limitations: First, the sample size was relatively modest, and some imbalance in demographic characteristics may limit the generalizability of the findings, despite statistical control for age and sex. Second, lesion location and overlap with speech-related regions were not systematically analyzed, as the present study focused on surface-based cortical morphology rather than lesion–symptom mapping. In particular, subgroup analyses comparing supratentorial and infratentorial stroke patients may have been underpowered. Second, the cross-sectional design precludes assessment of longitudinal cortical changes and limits inference regarding the temporal relationship between morphological alterations and functional recovery. Longitudinal studies are needed to clarify how cortical surface features evolve during post-stoke rehabilitation. Third, although SBM provides sensitive measures of cortical structure, it primarily captures cortical features and maty be less sensitive to subcortical, brainstem, or cerebellar pathology. In addition, SBM results can be influenced by image quality and methodological choices in data processing. Finally, although the correlations between cortical gyrification of the right supramarginal gyrus and FDA scores reached statistical significance, the effect sizes were modest. This suggests that single cortical morphological metrics are unlikely to fully explain speech impairment severity or recovery at the individual level. Instead, these measures may serve as potential imaging markers that contribute to a broader, multimodal understanding of post-stroke dysarthria. Clinical assessment relied mainly on the FDA. While widely used, this scale may not fully capture all aspects of speech and swallowing function. Future studies integrating more detailed behavioral measures and multimodal imaging techniques, such as functional MRI or diffusion imaging, may provide a more comprehensive understanding of the relationship between structural changes and functional outcomes in post-stroke dysarthria.

## Data Availability

The original contributions presented in the study are included in the article/supplementary material, further inquiries can be directed to the corresponding authors.

## Footnotes

1
http://dbm.neuro.uni-jena.de/cat/
